# Identification and expression analysis of BURP domain-containing genes in jujube and their involvement in low temperature and drought response

**DOI:** 10.1186/s12864-022-08907-9

**Published:** 2022-10-06

**Authors:** Wenzhu Wang, Zhong Zhang, Xingang Li

**Affiliations:** 1 College of Forestry, Research Center for Jujube Engineering and Technology of State Forestry Administration, Northwest A&F University, Yangling, 712100 Shaanxi China; 2 Agricultural Genomics Institute at Shenzhen, Chinese Academy of Agricultural Sciences, Shenzhen, 518116 Guangdong China

**Keywords:** *Ziziphus jujuba* Mill, BURP domain-containing protein, Gene expression, Abiotic stress, Fruit development

## Abstract

**Background:**

Plant-specific BURP domain-containing genes are involved in plant development and stress responses. However, the role of *BURP* family in jujube (*Ziziphus jujuba* Mill.) has not been investigated.

**Results:**

In this study, 17 *BURP* genes belonging to four subfamilies were identified in jujube based on homology analysis, gene structures, and conserved motif confirmation. Gene duplication analysis indicated both tandem duplication and segmental duplication had contributed to *ZjBURP* expansion. The *ZjBURPs* were extensively expressed in flowers, young fruits, and jujube leaves. Transcriptomic data and qRT-PCR analysis further revealed that *ZjBURPs* also significantly influence fruit development, and most genes could be induced by low temperature, salinity, and drought stresses. Notably, several *BURP* genes significantly altered expression in response to low temperature (*ZjPG1*) and drought stresses (*ZjBNM7*, *ZjBNM8*, and *ZjBNM9*).

**Conclusions:**

These results provided insights into the possible roles of *ZjBURPs* in jujube development and stress response. These findings would help selecting candidate *ZjBURP* genes for cold- and drought-tolerant jujube breeding.

**Supplementary Information:**

The online version contains supplementary material available at 10.1186/s12864-022-08907-9.

## Background

Plants suffer diverse abiotic and biotic stresses, such as low temperature, drought, soil salinity, and damage from several diseases and pests, during their lifecycle. Stress influenced plant growth, development, and qualities in varying degree. In contrast, plants have also evolved certain mechanisms to settle in disadvantageous condition. Unraveling the mechanism of plant response to stress would be helpful for crops and fruit trees to gain knowledge needed for maintaining the agriculture and food security with increasing world population [1]. Recently, progresses have been made to elucidate plant response by identifying stress-related genes, metabolites, and pathways [2]. Some studies proved that a stress-responsive gene family encoding BURP domain-containing protein is plant-specific and performs important functions in plant development and stress response [3, 4]. The BURP proteins contain a BURP domain with conserved structures of CHX_10_CHX_23–37_CHX_23–26_CHX_8_W [5] and has been identified in some plant species, including rice (*Oryza sativa*) [6], soybean (*Glycine max*) [7], maize (*Zea mays*) [8], poplar (*Populus trichocarpa*) [9], cotton (*Gossypium raimondii*, *Gossypium arboretum*, and *Gossypium hirsutum*) [10], common bean (*Phaseolus vulgaris*) [11], Chinese rose (*Rosa chinensis*) [12], and legumes (*Phaseolus vulgaris*, *Cicer arietinum*, *Cajanus cajan*, and *Vigna radiata*) [13].

The BURP family is classified into BNM2-like, USP-like, RD22-like, and PG1β-like subfamilies; moreover, other subfamilies specifically exist in few species. Members belonging to different subfamilies has varied expression and function to maintain plant development. For instance, *BnBNM2* is expressed in *Brassica napus* during microspore embryogenesis [14–16]. *VfUSP* participates in the early development of zygotic embryogenesis of field bean [17, 18]. Additionally, anther-specific BURP proteins, *OsRAFTIN1* and *RA8*, were both important for anther dehiscence in rice [4, 19]. Moreover, *SCB1* extensively affects seed coat formation [20]. Moreover, BURP proteins have also displayed their critical functions in fruit development and ripening. For example, *VvBURP1* is involved in early fruit morphogenesis of *Vitis vinifera* [21], and *PG1*β monitors pectin solubilization and degradation during tomato maturation [22, 23].

The *BURP*s may be also induced by phytohormones and abiotic stress treatments, including abscisic acid (ABA), salicylic acid (SA), NaCl, cold, and drought. For instance, *AtUSPL1*, belonging to *RD22* subfamily, suppresses ABA-mediated drought stress response [24]. *BgBDC1*, *2*, *3*, and *4* also have similar responses to salt and drought in an ABA-mediated pathway in mangroves [25]. Besides, both ABA and SA may induce all *RD22*-like *GhBURPs* in cotton [10]. Additionally, *OsBURPs* and most *PtBURPs* are significantly expressed in *Oryza sativa* and poplar, respectively, in response to at least one stress treatment, including cold, salt, drought, and ABA. These results indicated the potential role of *BURP*s in plant stress. However, the function of these *BURPs* remains to be further investigated. We have previously identified two *BURP* genes possibly associated with the variation of several cyclopeptide alkaloids in 214 jujube (*Ziziphus jujuba* Mill.) accessions using mGWAS analysis [26]. Both *BURP* genes showed consistent expression along with the metabolite accumulation. Therefore, the BURP domain-containing genes in jujube genome should be identified and investigate their potential roles in jujube development and stress response.

Jujube is an economically and ecologically important fruit tree with immense edible and medicinal values [27, 28]. Moreover, jujube is highly adaptative and tolerant to adverse environmental conditions, and wild jujube colonizes barren mountains. Thus, as jujube is a pioneer tree, revealing and studying its stress-responsive candidate genes is of vital importance. Till date, the involvement of several transcription factors, such as *WRKY* [29], *NAC* [30], and *AP2/ERFs* [31], have been identified in stress response and fruit development in jujube, while few downstream functional protein gene families have been investigated [32, 33]. This study reports a functional protein gene family in jujube and attempts to understand their variation and candidate functions using a series of bioinformation analysis. *ZjBURP* transcript expression in different tissues during development processes and in jujube leaf under various stress conditions were further studied. Our findings provide an important foundation for future functional studies of *BURP* genes in jujube, and will be potentially useful for jujube molecular breeding to improve stress resistance and fruit quality, particularly in cold- and drought-tolerant jujube breeding.

## Results

### Identification, chromosomal location, and gene duplication of *BURPs* in jujube

A total of 17 putatively encoded *BURP* genes were identified from jujube genome. These genes were designated according to their phylogenetic relationship and previously reported homologous groups in other species. Notably, our result corrected a false induced by structural annotation in jujube reference genome, and therefore we manually identified two gene sequences as evm.model.Contig91.143 (*ZjPG2*) and evm.model.Contig91.146 (*ZjPG3*) from the genomic region. The information of 17 candidate genes, including their amino acid length, molecular weight (Mw), isoelectric point (pI), subcellular localization, and physical locations on chromosome, is summarized in Table 1. The length of ZjBURP protein sequences ranged from 215 (ZjBURP5) to 634 (ZjPG2 and ZjPG3). The Mw of ZjBURP proteins were between 24.08658 (ZjBURP5) and 70.05929 (ZjPG3), and the pI values ranged from 5.25 (ZjBURP4) to 8.93 (ZjBURP1).

**Table 1:** Summary of sequence bioinformation analysis of 17 *BURP* genes identified in jujube

Annotation ID	Gene Name	Length of protein sequence	Molecular weight/kDa of protein	Theoretical pI	Subcellular localization	Chromosome	Location
evm.model.Contig42.206	*ZjBNM1*	247	27.68268	6.02	Plasma Membrane(1.734) /Extracellular(1.173)	6	9081302–9082045( +)
evm.model.Contig42.207	*ZjBNM2*	251	27.74872	6.29	Cytoplasmic	6	9048619–9049374( +)
evm.model.Contig42.204	*ZjBNM3*	268	29.71128	6.48	Extracellular	6	9149419–9150828( +)
evm.model.Contig42.195	*ZjBNM4*	268	29.75033	6.29	Extracellular	6	9462264–9463207( +)
evm.model.Contig42.200	*ZjBNM5*	268	29.68431	6.24	Extracellular(1.373) /Cytoplasmic(1.013)	6	9277149–9278091( +)
evm.model.Contig42.209	*ZjBNM6*	337	38.48951	6.3	Extracellular(1.822) /Nuclear(1.361)	6	8952873–8954741( +)
evm.model.Contig19.168	*ZjBNM7*	289	32.89871	6.38	Plasma Membrane	2	5586889–5587918( −)
evm.model.Contig19.151	*ZjBNM8*	287	32.70449	6.1	Plasma Membrane(1.316) /Cytoplasmic(1.110) /Extracellular(1.003)	2	5779768–5780791( −)
evm.model.Contig19.147	*ZjBNM9*	287	32.71256	6.1	Plasma Membrane(1.395) /Cytoplasmic (1.124)	2	5845666–5846689( −)
evm.model.Contig70.221	*ZjBURP1*	330	35.44262	8.93	Extracellular	10	1655503–16656996( −)
evm.model.Contig78.1.67	*ZjBURP2*	312	35.29459	8.75	Extracellular	5	20910272–20911766( −)
evm.model.Contig78.1.57	*ZjBURP3*	319	35.92348	5.85	Extracellular	5	20848839–20851320( +)
evm.model.Contig78.1.60	*ZjBURP4*	217	24.69425	5.25	Extracellular	5	20867160–20867882( +)
evm.model.Contig78.1.59	*ZjBURP5*	215	24.08658	8.5	Nuclear	5	20859635–20860456( +)
evm.model.Contig58.93	*ZjPG1*	617	67.688	8.2	Extracellular	7	6542722–6544575( +)
evm.model.Contig91.143	*ZjPG2*	634	69.79507	8.73	Extracellular(1.749) /Vacuole(1.067)	2	13842223–13844585( −)
evm.model.Contig91.146	*ZjPG3*	634	70.05929	8.62	Extracellular	2	13850183–13852336( −)

**Fig. 1:**
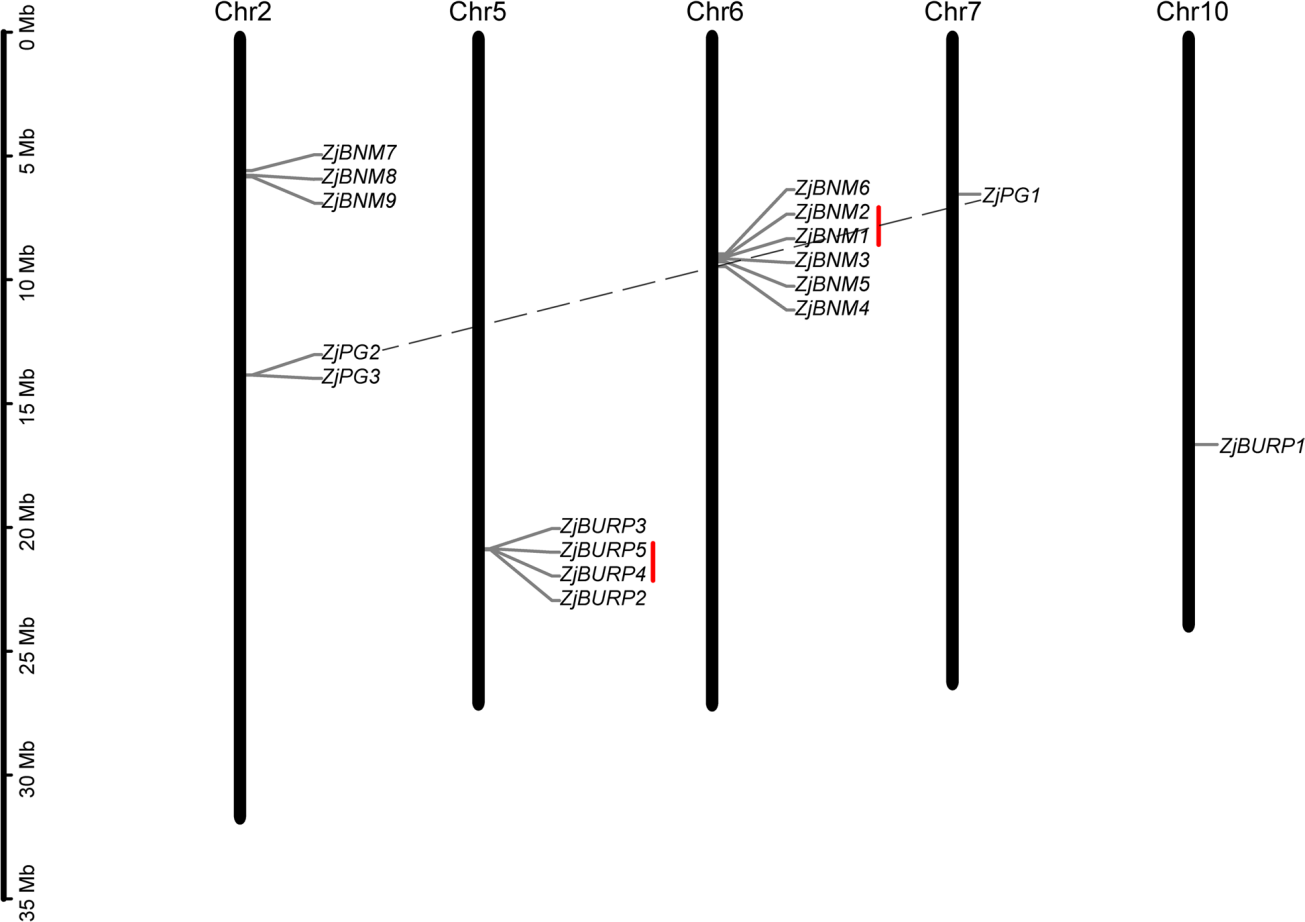
Chromosomal location and gene duplication events of *ZjBURPs*. The scale bar is in million bases (Mb). The genes connected with dotted gray and red lines represent the segmental (gray) and tandem (red) gene pairs, respectively

The 17 *ZjBURPs* were unevenly located on five chromosomes (Table 1 and Fig. 1). A total of 15 *BURPs* located on chr 2, 5, and 6 and 1 *ZjBURP* was anchored on chr 7 and 10. Interestingly, six genes (*ZjBNM1-6*) showed close distribution on chr 6, and four genes (*ZjBURP2-5*) were also located near each other on chr 5. We further performed gene duplication analysis and demonstrated that two tandem duplication events (*ZjBURP4* / *ZjBURP5*, *ZjBNM1* / *ZjBNM2*) and one segment duplication event (*ZjPG1* / *ZjPG2*) existed in nearby clusters.

### Phylogenetic analysis and classification of the *BURP* gene family in jujube

**Fig. 2:**
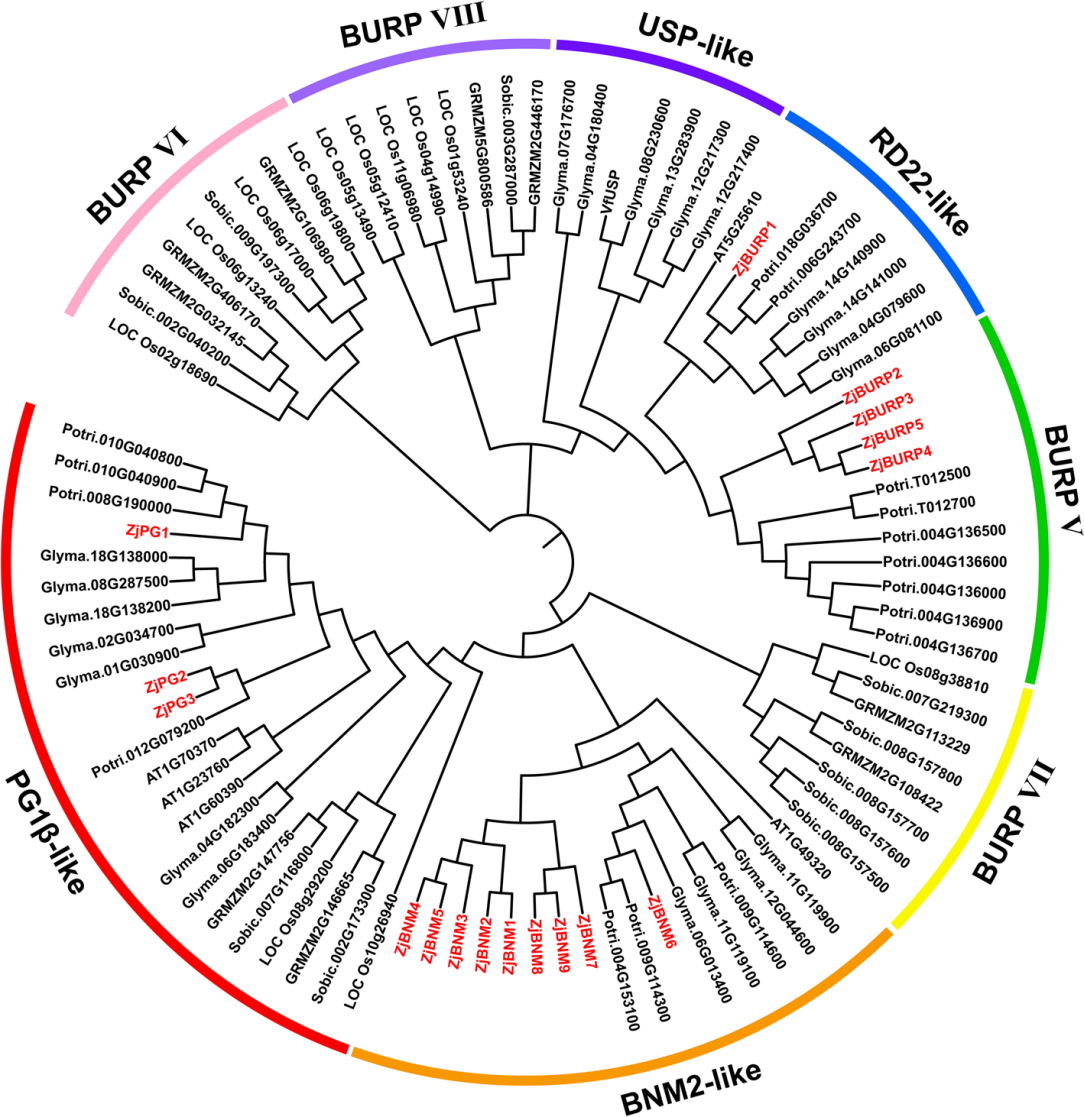
The phylogenetic tree of BURP proteins and *ZjBURP* classification. The protein sequences from *Brachypodium distachyon* (10), *Setaria italica* (13), *Oryza sativa* (12), *Sorghum bicolor* (10), *Zea mays* (9), *Arabidopsis thaliana* (5), *Glycine max* (21), *Cucumis sativus* (6), *Citrus sinensis* (6), *Brassica rapa* (9), *Populus trichocarpa* (16), *Vicia faba* (1), and *Ziziphus jujuba* (17) were aligned using MUSCLE algorithm in MEGA-X, and the phylogenetic tree was constructed by MEGA-X with the neighbor-joining (NJ) method and 1,000 bootstrap replicates

To explore the evolutionary relationships of BURP proteins, a phylogenetic tree was generated by clustering 135 BURP proteins from different species, which were then classified into eight distinct subfamilies (BNM2- like, USP-like, RD22-like, PG1β-like, BURP V, BURP VI, BURP VII, and BURP VIII; Fig. 2). All 17 *ZjBURPs* belonged to four subfamilies, including BNM2-like, RD22-like, PG1β-like and BURP V.

Additionally, BNM2-like subfamily contained nine genes (*ZjBNM1–9*) adjacent to each other in the phylogenetic tree except *ZjBNM6*. BURP V subfamily consisted of four genes (*ZjBURP2–5*), which were also closely located. Two genes of PG1β-like subfamily, *ZjPG2* and *ZjPG3*, were close except *ZjPG1*. The relative position of 17 genes in phylogenetic tree were extremely similar to the distribution of them on chromosomes, suggesting that the gene locations on chromosomes might be consistent with their evolutionary relationship.

### Sequence alignment of BURP proteins in jujube

The signal peptides and BURP domains of these proteins in jujube were detected. The results showed that 17 and 12 BURP proteins contained the BURP domain and signal peptides, respectively (Fig. S1). Moreover, BURP proteins with close evolutionary relationships showed similar signal peptide and BURP domain composition and arrangement. The multiple sequence alignment analysis of these proteins revealed the existence of many highly conserved amino acid residues and four CH motifs. The results indicated that they were vital for the basic function of BURP family in jujube. Moreover, the C-terminus of BURP proteins in jujube could be concluded as CHX_5_YX_4_CHX_25–34_CHXDTX_2_WX_6_FX_2_LX_4_GX_3_ CHX_8_W with more conserved sequences between the first two CH motifs (Fig. S2).

### Gene structure and conserved motifs of *BURPs* in jujube

**Fig. 3:**
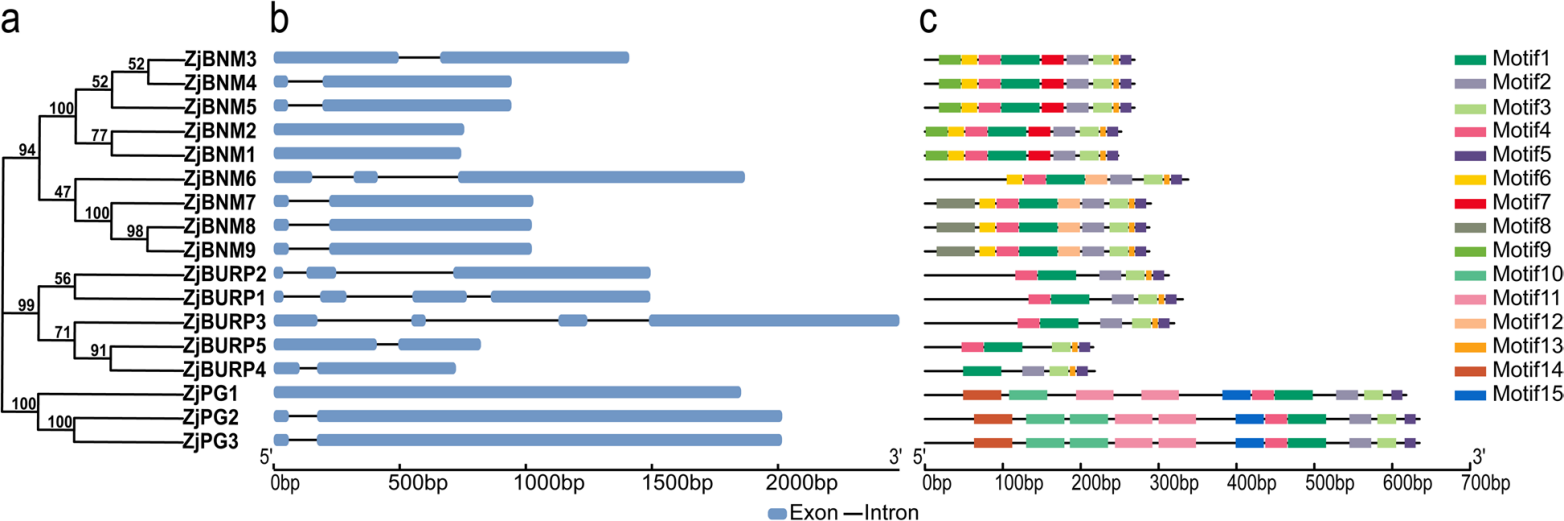
The phylogenetic relationships, gene structure, and conserved motifs of *ZjBURPs. ***a** The construction of the phylogenetic tree with the neighbor-joining method. **b** *ZjBURP* gene structure. **c** The conserved motifs identified in *ZjBURP* gene family

To investigate *BURP* conservation and diversification in jujube, gene structures and conserved motifs were further analyzed (Fig. 3). Most *ZjBURPs* contained at least one intron, except *ZjBNM1*, *ZjBNM2*, and *ZjPG1*.

Most members from BNM2-like and PG1β-like subfamilies contained two exons, whereas genes from RD22-like and BURP V subfamilies contained 2–4 exons. These results showed that members from the same subfamily owned similar gene structure (Fig. 3a, b).

We further detected 15 conserved motifs utilizing MEME to explore the similarity and differentiation of BURP proteins in different subfamilies (Fig. 3c). The BURP domain in C-terminus contained motifs 4, 1, 8, 3, 5. Besides, five genes (*ZjBNM1-5*) from BNM2-like subfamily also contained motif 7, and all genes contained motif 13 in the C-terminus except those belonging to PG1β-like subfamily. Notably, motifs 6, 8, and 9 and motifs 10, 11, 14, and 15 existed only in BNM2-like and PG1β-like subfamilies, respectively. Overall, motif compositions and arrangements were particularly similar in *ZjBURPs* with close relationship.

### Analysis of *cis*-elements in *ZjBURP* promoter regions

**Fig. 4:**
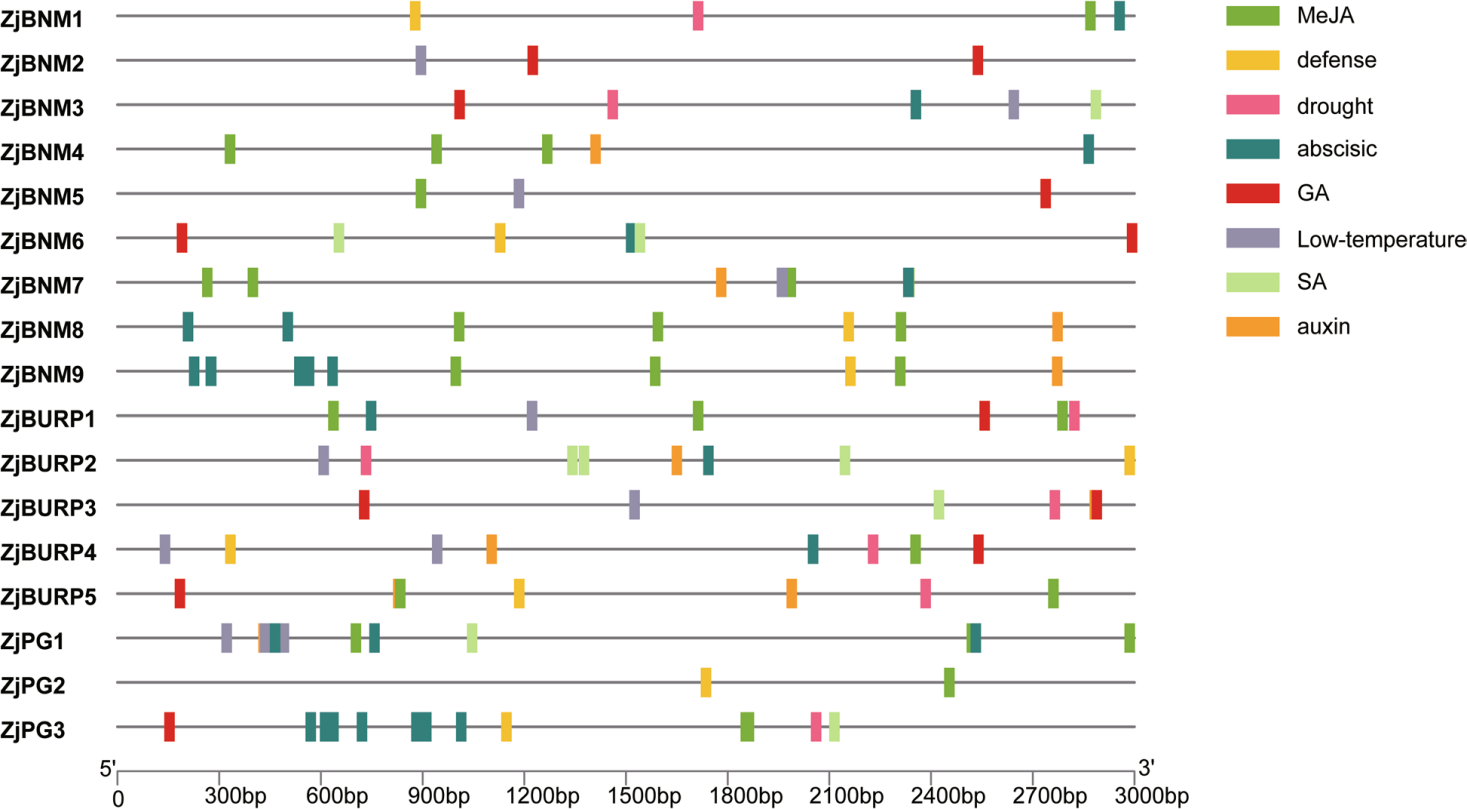
Distribution of stress- and phytohormone-related *cis*-elements in the putative *ZjBURP* promoter region*.* The location of these *cis*-elements was predicted by plantCARE database

To investigate the possible regulatory functions of *cis*-elements in the putative promoter regions of *ZjBURPs*, stress- and phytohormone-related *cis*- elements in the 3,000 bp upstream of the start codons in all 17 *ZjBURPs* were identified (Fig. 4 and Table S1). The identified phytohormone-related elements were AuxRR-core (auxin), TGA-element (auxin), ABRE (ABA), P-box (gibberellin), TATC-box (gibberellin), GARE-motif.

(gibberellin), CGTCA-motif (MeJA), TGACG-motif (MeJA), and TCA-element (SA). Stress-responsive regulatory elements included MBS (drought), TC-rich repeats (defense and stress responsiveness), and LTR (cold stress). All *ZjBURPs*, expect *ZjBNM4*, contained at least one putative stress-related elements. Moreover, the promoters of some *ZjBURPs*, such as *ZjBURP4* (1 MBS, 2 LTRs and 1 TC-rich repeats) and *ZjBURP2* (1 MBS, 1 LTR and 1 TC-rich repeats), contained multiple stress-responsive elements. Notably, low temperature-related elements were more common than drought- and defense-related elements, and *ZjPG1* owned up to three low temperature elements. All members from RD22-like and BURP V subfamilies contained one drought-responsive element. In addition, ABA- and MeJA-related elements were more general than other phytohormone-related elements, indicating that *ZjBURPs* might take part in the ABA or MeJA regulatory pathway.

### Transcript expression of *ZjBURPs* in different tissues during development

**Fig. 5:**
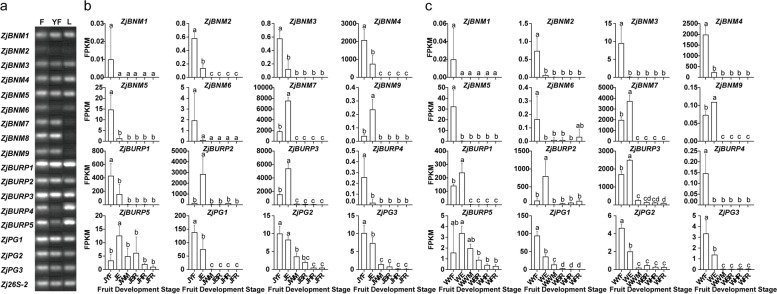
*ZjBURP* expression in three tissues and during fruit development processes. **a** RT-PCR analysis of *ZjBURPs* in three tissues. F: flower; YF: young fruit; L: leaf. **b** Transcriptional abundance of *ZjBURPs* during fruit development of ‘Junzao’. FPKM was selected to determine *ZjBURP* expression levels. JYF, JE, JWM, JBR, JHR, and JFR represent the young fruit, enlargement, white maturity, beginning-red, half-red, and full- red stages of ‘Junzao,’ respectively. **c** Transcriptional abundance of *ZjBURPs* during fruit development of ‘Qingjiansuanzao’. WYF, WE, WWM, WBR, WHR, and WFR represented the young fruit, enlargement, white maturity, beginning-red, half-red, and full-red stages of ‘Qingjiansuanzao,’ respectively

To gain insight into the potential function of *ZjBURPs* during jujube development, we estimated their. transcription level in different jujube tissues, including flowers, young fruit, and leaf by RT-PCR. The results showed that all *ZjBURPs* had various expression levels in three tissues (Fig. 5a, Fig.S3). Four genes *(ZjBURP1*, *2*, *3*, and *ZjPG1*) expression was high in all tissues (Fig. 5a). However, *ZjBNM2* expression was extremely low in tested tissues, suggesting that it may be a redundant gene or pseudogene, or expressed only in specific organs or at specific developmental processes or under specific conditions. In addition, three BNM2-like subfamily members (*ZjBNM7*, *8*, and *9*) expression in reproductive organs, like flowers and young fruit, was higher than that in leaves, implying their possible roles in fruit development. Besides, *ZjBURP4* showed dominant expression in leaves.

We further analyzed the *ZjBURP* expression levels during wild (Qingjiansuanzao) and cultivated jujube (*Z. jujuba* cv. Junzao) fruit development with in-house transcriptomic data to investigate their potential role in fruit development. *ZjBNM8* was not expressed in either wild or cultivated jujube fruit (Fig. 5b, c). The results revealed that 16 *ZjBURPs* showed similar expression patterns, with preferential expression at young fruit and enlargement between the two accessions during fruit development, which indicated that *ZjBURPs* may play important roles in young fruit development and enlargement in both wild and cultivated jujube. Furthermore, transcript abundance at young fruit and enlargement varied among these genes, and six genes (*ZjBNM4*, *ZjBNM7*, *ZjBURP1*, *ZjBURP2*, *ZjBURP3*, and *ZjPG1*) had higher transcript abundance (FPKM ≥ 30) than other genes.

### *ZjBURP* expression in wild jujube leaf in response to different stress conditions

Based on the analysis of *cis*-elements in promoter regions and previous reports on *BURP* genes in response to diverse stresses in other plants, *ZjBURPs* might be stress-responsive in jujube. To examine whether *ZjBURPs* are involved in various stresses, the transcript levels of all *ZjBURPs* were investigated in the leaves of wild jujube seedlings under three treatments including low temperature, salt, and drought stresses.

**Fig. 6:**
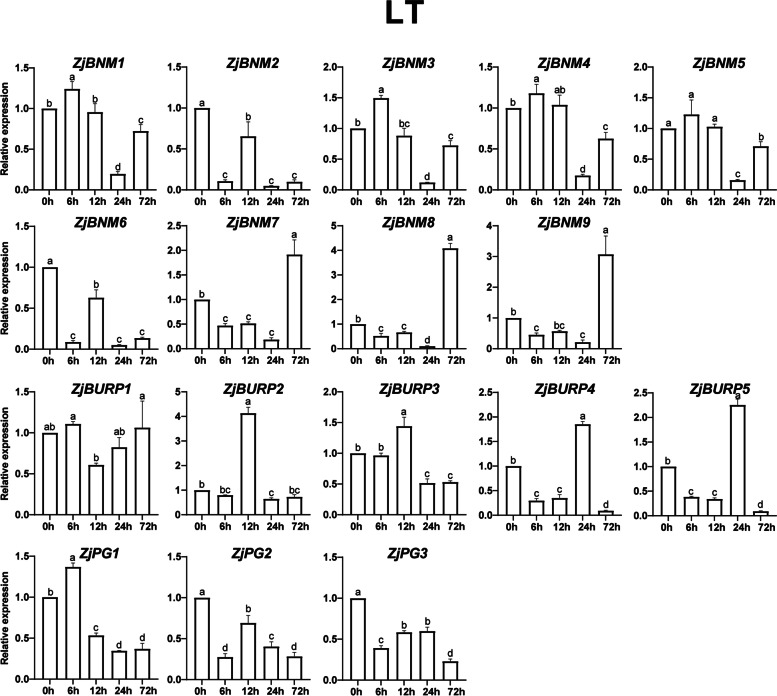
*ZjBURP* expression in low temperature. 0 h, 6 h, 12 h, 24 h, and 72 h represent the time period after the treatment. The error bars indicate the standard deviation of three biological replicates. Different letters represent a significant variation between the experimental groups (*p* < 0.05)

All *ZjBURPs* were more or less upregulated or downregulated under low temperature (4 ℃), revealing the possible roles of *ZjBURPs* in response to cold stress (Fig. 6). Furthermore, two main expression patterns were found. One expression pattern included the downregulation of *ZjBURPs*, including *ZjBNM2*, *ZjBNM6*, *ZjPG2*, and *ZjPG3*, at all timepoints after cold treatment. The other expression pattern involved the upregulation of *ZjBURPs* at certain timepoints and downregulation at other timepoints, and it could be divided into five categories: upregulation at 6 h, including *ZjBNM1*, *ZjBNM3*, *ZjBNM4*, *ZjBNM5*, and *ZjPG1*; upregulation at 12 h, containing *ZjBURP2* and *ZjBURP3*; upregulation at 24 h, comprising *ZjBURP4* and *ZjBURP5*; upregulation at 72 h, consisting of *ZjBNM7*, *ZjBNM8*, and *ZjBNM9*; upregulation at 6 h and 72 h, exemplified by *ZjBURP1*. The results showed that the differential expression characteristics of *ZjBURPs* existed also in the same subfamily, and those genes with close relationship might share similar expression patterns.

**Fig. 7:**
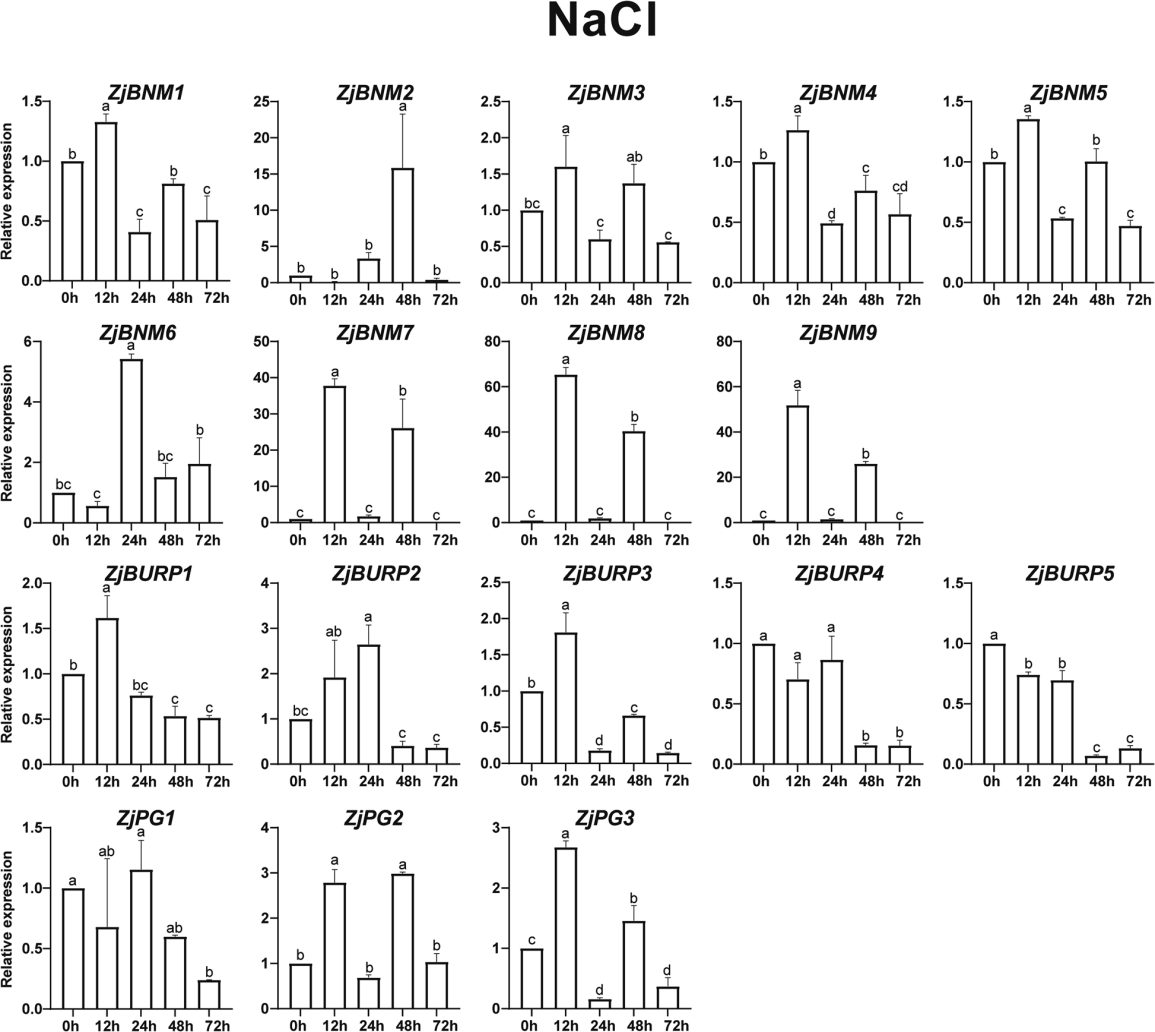
*ZjBURP* gene expression under NaCl treatment. 0 h, 12 h, 24 h, 48 h, and 72 h represent the time period after NaCl treatment. The error bars indicate the standard deviation of three biological replicates. Different letters represent a significant variation between the experimental groups (*p* < 0.05)

Under salt stress condition, only two genes (*ZjBURP4* and *ZjBURP5*; both belonging to BURP V subfamily) were downregulated, whereas the remaining 15 genes were upregulated with different degrees and various expression characteristics (Fig. 7). Among the upregulated genes, four expression patterns were observed. One was upregulation at 12 h and then downregulation, such as that for *ZjBNM1*, *ZjBNM3*, *ZjBNM4*, *ZjBNM5*, *ZjBURP1*, and *ZjBURP3*. The second expression pattern was upregulation at 12 h and 48 h, such as that for *ZjBNM7*, *ZjBNM8*, *ZjBNM9*, *ZjPG2*, and *ZjPG3*. The third expression pattern was upregulation at 24 h, such as that for *ZjBNM6*, *ZjBURP2*, and *ZjPG1*. *ZjBNM2* represented the last expression pattern, with upregulation at 48 h. The results revealed that all *ZjBURPs* could be induced by salt stress; notably, *ZjBNM8* expression level was the highest, with over 60-fold upregulation.

**Fig. 8:**
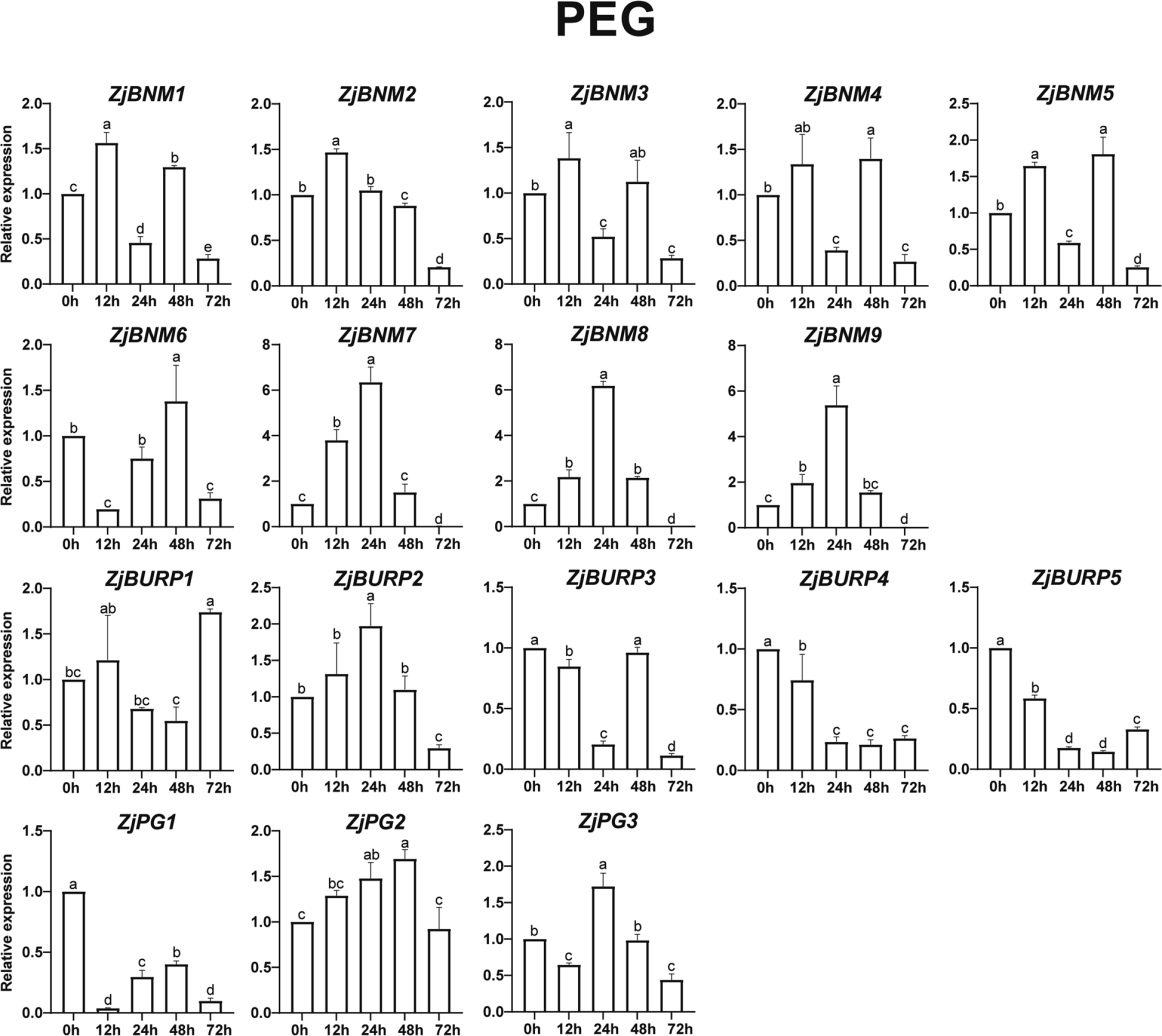
*ZjBURP* gene expression under PEG treatment. 0 h, 12 h, 24 h, 48 h, and 72 h represent the time period after PEG treatment. The error bars indicate the standard deviation of three biological replicates. Different letters represent a significant variation between the experimental groups (*p* < 0.05)

Four genes (*ZjBURP3*, *ZjBURP4*, *ZjBURP5* and *ZjPG1*) were downregulated, while other genes were upregulated in seedlings under drought stress (Fig. 8). The other 13 *ZjBURPs* exhibited four expression patterns. One was upregulation of *ZjBURPs* at 12 h and 48 h, including *ZjBNM1*, *ZjBNM2* (slight downregulation at 24 h), *ZjBNM3*, *ZjBNM4*, and *ZjBNM5*. The second expression pattern was significant upregulation at 24 h, such as that for *ZjBNM7*, *ZjBNM8*, *ZjBNM9*, *ZjBURP2*, and *ZjPG3*. The third expression pattern was upregulation at 48 h, such as that for *ZjBNM6* and *ZjPG2*. The last expression pattern was specific for *ZjBURP1*, with upregulation at 72 h. Among these genes, three genes (*ZjBNM7*, *ZjBNM8*, and *ZjBNM9*). were mostly induced approximately six-folds after 24 h drought stress, indicating they might perform significant functions in response to drought stress in jujube. We also noticed that six genes (*ZjBNM1*, *ZjBNM3*, *ZjBNM4*, *ZjBNM5*, *ZjBURP2*, and *ZjBURP5*) exhibited similar expression characteristics under salt and drought stress.

## Discussion

*BURP* genes have been extensively analyzed in several plants for their significant roles in plant development and stress response [18, 22, 23, 34]. However, this gene family has not been studied in jujube. This study reports a comprehensive analysis of the *BURP* gene family in jujube, including analysis of chromosomal location, gene duplication events, phylogeny, sequence alignment, gene structure, conserved motifs, *cis*-elements in the promoter region, and expression analysis. A total of 17 *BURP* genes were identified in ‘Junzao’ genome, and the variation of length of these sequences suggested high complexity within the *ZjBURP* gene family, as also reported in *Medicago* [35]. In addition, the genome sequences of ‘Dongzao’ and ‘Wild jujube’ have also been reported recently [36, 37]; therefore, we can obtain more insight into the synteny and evolution of *BURP* gene family among wild, fresh, and dry jujube. Moreover, the chromosomal location of *ZjBURPs* in ‘Junzao’ genome may also provide clues for the formation and evolution of this gene family in jujube. Four *BURP* gene clusters were distributed on three jujube chromosomes, and genes belonging to the same cluster might originate from a common ancestor and formed through gene duplication. Further analysis of gene duplication events showed that two and one *BURP* gene pairs were involved in tandem and segmental duplication events, respectively, indicating that both tandem and segmental duplication events served as the vital driving force during the long-term of *BURP* gene evolution in jujube. Similar results have been observed in *G. raimondii* and *G. hirsutum* [10]; however, in many plants, only one gene duplication event occurred in *BURP* gene family, such as one tandem duplication in *G. arboretum* [10], six tandem duplication events in *Medicago* [35], and one segmental duplication event in *R. chinensis* [12]. These results implied that *BURP* genes are subjected to strong positive selection in numerous plants, and conformed to different expansion models in diverse plants [38].

According to the phylogenetic tree, the *BURP* members from 13 plants could be divided into eight subfamilies: BNM2-like, USP-like, RD22-like, PG1β-like, BURP V, BURP VI, BURP VII, and BURP VIII. Interestingly, the *BURP* genes belonging to BNM2-like, USP-like, and BURP V subfamilies were only found in investigated dicotyledonous species (dicots), whereas those belonging to the BURP VI and BURP VII subfamilies contained only the BURP members from investigated monocotyledonous species (monocots). These results were consistent with the results of many previous studies [10, 38]. The PG1β-like subfamily was observed in both dicots and monocots, indicating that PG1β-like subfamily might have originated before the divergence of monocot and dicot plants, and BNM2-like, USP-like, as well as BURP V subfamilies, might have subsequently evolved separately and exert specific functions in dicots. Some results were distinguished from those of previous studies due to the number of species analyzed in the phylogenetic analysis for more reliable results of classification. Notably, BURP proteins from cotton, poplar, and jujube showed similar distributions in four subfamilies (BNM2-like, RD22-like, PG1β-like and BURP V).

The further structural analysis showed that all 17 BURP proteins in jujube included the conserved BURP domain, particularly the four CH motifs, which were also detected in BURP sequences in previously studied plants, like cotton [10], rose [12], *Medicago* [35], and rice [6], hinting that they may be crucial for the basic function of BURP proteins. Moreover, the component motifs and their arrangement in *ZjBURPs* were conserved within a subfamily and different in various subfamilies, which correspond to the results of the structure analysis of *ZjBURPs*. Similar findings were obtained in previous studies and might be relative with the conserved and diverse functions of *BURP* genes [7, 8].

To elucidate the possible functions of *ZjBURPs* in jujube development, their expression in different organs during development processes were analyzed. The results of expression patterns in three organs suggested that all *ZjBURPs* were expressed in flowers, young fruits and leaves, and some *ZjBURPs* with high sequence similarity also exhibited similar expression characteristics. For instance, *ZjPG2* and *ZjPG3*, which shared similar sequences, were both transcribed in all three organs and highly expressed in flowers. Three closely clustered genes, *ZjBNM7*, *ZjBNM8* and *ZjBNM9*, were highly expressed in flowers and young fruits, and weakly expressed in leaves. However, not all genes with close relationship had the same expression pattern, such as *ZjBURP4* and *ZjBURP5*, as gene duplication pairs, showed different expression characteristics. *ZjBURP4* expression was largely restricted to the leaves, whereas *ZjBURP5* was also transcribed in flowers. The conserved and diversification of *BURP* expression patterns have also been observed in soybean [7]. Notably, *ZjBNM2* transcript levels in all three tissues were extremely low, however, the degree of its upregulation under NaCl treatment was significant, indicating that it was induced by salt stress and its expression levels might be suppressed under normal conditions. Furthermore, the results of *BURP* expression patterns during fruit development showed that all 17 *ZjBURPs* were preferentially expressed during young fruit development and fruit enlargement in both ‘Junzao’ and ‘Qingjiansuanzao,’ indicating that *ZjBURPs* may execute important functions in young fruit development and enlargement, which was supported by the results of a previous study on a BURP protein associated with grapevine fruit development [21, 39]. In particular, several *ZjBURPs* were strikingly highly expressed (over 400- and 200-fold) during fruit development and enlargement, respectively. For instance, *ZjBNM7* is homologous to *AtUSPL1*, which associates with the seed development [40]; *ZjBURP1*, *ZjBURP2*, and *ZjBURP3* shared similar sequence with *GhRDL1* in cotton, which is predominantly transcribed during fiber rapid elongation and promotes fiber elongation by interacting with *GhEXPA1* [41]; *ZjPG1* is homologous to *AtPGL3*, which promotes cell enlargement [42]; these *ZjBURPs* were highly expressed during young fruit development and fruit enlargement, indicating their key roles in regulating jujube development and enlargement, which should be verified in future functional analysis.

Besides, *BURP* genes are also pervasively involved in plant stress response in many plants. To investigate whether *ZjBURPs* were similarly responsive to stresses, we analyzed their stress-related *cis*-elements in the promoter region using qRT-PCR under different stresses, including low temperature, salt, and drought. The analysis of *cis*-elements showed that all promoters of *ZjBURPs* contained at least two investigated stress-related elements, implying that these genes might respond to different stresses. Furthermore, qRT-PCR analysis reports that the transcription of all *ZjBURPs* were induced by cold, salinity, and drought stresses, although several genes were slightly induced. Under low temperature condition, *ZjPG2* and *ZjPG3* expression were significantly downregulated, which were similar to the expression pattern of *OsBURP12*, which belongs to the same subfamily, in rice [6]. Moreover, *ZjPG1* expression was similar to that of *OsBURP16*, which also belongs to the PG1β-like subfamily and decreases cold tolerance by enhancing PG activity and reducing the pectin content of cell wall in *OsBURP16*-transgenic plants [43, 44]. Therefore, we may conclude that *ZjPG1* downregulation is important for jujube cold tolerance. Consistently, *ZjPG1* contained the most low temperature-responsive *cis*-elements among all the *BURP* genes in jujube, indicating that it might be involved in jujube cold response through the low temperature-responsive *cis*-elements. Most *ZjBURPs*, except *ZjBURP4* and *ZjBURP5*, were upregulated under NaCl treatment. In particular, *ZjBNM7*, *ZjBNM8*, and *ZjBNM9* expression levels were elevated 12 h and 48 h after NaCl treatment, indicating that these genes could positively respond to salt stress. Under drought treatment, several *ZjBURPs*, including *ZjBNM1*, *ZjBNM3*, *ZjBNM4*, *ZjBNM5*, *ZjBURP4*, and *ZjBURP5*, possessed similar expression characteristics to those under salt stress. In addition, *ZjPG1* was downregulated under drought treatment, which was similar to *MtBURP33* and *MtBURP28* downregulation in *Medicago* [35]. *ZjBNM7*, *ZjBNM8*, and *ZjBNM9* expression levels were strikingly upregulated by drought stress, indicating that they might be key *BURP* genes involved in jujube drought response. This result was supported by their close relationship to *AtUSPL1*, which is upregulated as part of the ABA-mediated moisture stress response and involved in *Arabidopsis thaliana* drought tolerance [24, 34, 45, 46]. Interestingly, no drought-responsive *cis*-elements were found in their promoter regions, revealing that the results of qRT-PCR are not always consistent with *cis*-elements analysis [35], suggesting that many *BURP* genes with no related *cis*-elements identified in this study may have other regulation patterns.

## Conclusions

In this study, *BURP* genes in the jujube genome were systematically analyzed. The 17 identified *ZjBURPs* could be classified into four subfamilies based on phylogeny, gene structure and conserved motif analysis. Gene duplication analysis indicated that both tandem and segmental duplication events might contribute to the *ZjBURP* gene family expansion. Expression analysis showed that all *ZjBURPs* were transcribed in flowers, young fruits and leaves, and four genes (*ZjBURP1*, *ZjBURP2*, *ZjBURP3* and *ZjPG1*) were highly expressed in all three tissues. Transcriptome data revealed that *ZjBURPs* were preferentially expressed at young fruit and enlargement stages in both ‘Junzao’ and ‘Qingjiansuanzao,’ indicating their possible roles in regulating young fruit development and enlargement in jujube. The qRT-PCR analysis of *ZjBURP* genes under various stress treatments indicated that all *ZjBURPs* were induced by low temperature, salt and drought, even though some genes were slightly induced. We also concluded that several key *BURP* genes may perform significant functions in response to low temperature (*ZjPG1*) and drought stresses (*ZjBNM7*, *ZjBNM8*, and *ZjBNM9*), and their functions needs to be confirmed in future studies. This study provides a comprehensive view of the *ZjBURP* gene family and may serve as foundation for future breeding of cold/drought-tolerant jujube trees.

## Methods

### Plant material and treatment

Wild jujube seeds were collected from the Germplasm Resource Nursery (Xingtai, China) and sowed in sterilized soil after rinsing with tap water for 24 h. All seedlings were grown at 24 ℃ in a climate-controlled glasshouse (light/dark cycle: 16 h/8 h), and four-week-old seedlings with uniform height and biomass were selected for treatments. For salt and PEG6000-simulated drought stress treatment, seedlings were treated with 100 mM NaCl, 20% PEG6000, and water as control after one-day adaptation to the hydroponic conditions. The leaves were collected at 0, 12, 24, 48, and 72 h after treatments. For low temperature stress, seedlings were transferred to 4 ℃ and those grown at 24 ℃ served as a control group. The leaves were collected at 0, 6, 12, 24, and 72 h after treatment. All samples were immediately frozen in liquid nitrogen and transferred to − 80 ℃ until analysis.

### Identification, chromosomal location, and gene duplication of *BURP* genes in jujube

The BURP protein sequences of *Arabidopsis thaliana* were downloaded from Swissprot database (https://www.uniprot.org) and used as queries for local blast analysis. Jujube genome dataset was obtained from our previous publication [47]. The blast output results were further aligned by Protein BLAST in NCBI (https://blast.ncbi.nlm.nih.gov) and confirmed for BURP domain using Batch Web CD-Search Tool (https://www.ncbi.nlm.nih.gov/Structure/bwrpsb/bwrpsb.cgi). Next, the Mw and pI of these validated proteins were predicted using ExPASy website (http://web.expasy.org/protparam/) [48]. The subcellular localization was predicted by CELLO v2.5 server (http://cello.life.nctu.edu.tw/) [49]. Chromosomal distribution of *ZjBURPs* was visualized based on their physical location from jujube genome databases. Gene duplication events were determined by MCScanX [50, 51].

### Sequence alignment of *BURP* genes and phylogenetic analysis

All ZjBURP protein sequences were multi-aligned using DNAMAN v.10.3.3.126 (Lynnon Biosoft, CA, USA) [52]. In addition, BURP proteins from jujube and several other plants, including *Brachypodium distachyon*, *Setaria italica*, *Oryza sativa*, *Sorghum bicolor*, *Zea mays*, *Arabidopsis thaliana*, *Glycine max*, *Cucumis sativu*, *Citrus sinensis*, *Brassica rapa, Vicia faba*, and *Populus trichocarpa*, were multi-aligned by MEGA-X [38, 53]. A phylogenetic tree was further constructed through the neighbor-joining (NJ) method of MEGA-X with Poisson model and 1,000 bootstrap replications, and the results were finally visualized by IToL v6.5.2 (http://itol.embl.de) [54].

### *BURP* sequence analysis in jujube

Distribution of predicted signal peptides and BURP domains in jujube genes were analyzed using SignalP 4.0 server (http://www.cbs.dtu.dk/services/SignalP/) [55] and the Batch Web CD-Search Tool (https://www.ncbi.nlm.nih.gov/Structure/bwrpsb/bwrpsb.cgi), respectively. *ZjBURP* gene structures were illustrated by Gene Structure Display Server 2.0 (GSDS: http://gsds.cbi.pku.edu.cn/) [56]. The online MEME program [57] was employed to detect the conserved motifs of ZjBURP proteins with following parameters: size distribution, zero or one occurrence per sequence; motif count, 15; and motif width, 6–50 amino acids. The *cis*-elements in the region of 3,000 bp upstream of the transcription initiation site of *ZjBURP* genes were predicted by PlantCARE database (http://bioinformatics.psb.ugent.be/webtools/plantcare/html/) [58].

### Transcript expression of *ZjBURPs* by RT-PCR and qRT-PCR

*ZjBURP* expressions in different organs, including flowers, young fruit, and leaves of jujube were analyzed by RT-PCR. Total RNA was isolated from samples using *SteadyPure* Plant RNA Extraction Kit (Accurate Biotechnology; Hunan, China). RNA quality and integrity was confirmed utilizing a NanoDrop 2000 UV–vis spectrophotometer (Thermo Fisher Scientific, USA) and 1% agarose gel electrophoresis, respectively. The RNA was reversed using *Evo M-MLV* RT Premix (Accurate Biotechnology). The gene-specific primers for 17 *ZjBURPs* and *Zj26S-2* (reference gene) were designed by Primer Premier 5 (Table S2). RT-PCR was carried out using 2 × Rapid Taq Master Mix (Vazyme) on an ABI 2720 Thermal Cycler (ABI; Marsiling, Singapore), and PCR products were detected by 1% agarose electrophoresis.

In addition, the relative expression levels of *ZjBURPs* in wild jujube leaf in response to different stresses (low temperature, salt and drought stresses) were determined by qRT-PCR using SYBR® Green Premix *Pro Taq* HS qPCR Kit II AG11702 on a Roche LightCycler96. The protocol was set as follows: 95 °C for 30 s; 40 cycles of 95 °C for 5 s, 58 °C for 30 s, 72 °C for 30 s; and 95 °C for 10 s, 65 °C for 60 s, 97 °C for 1 s; 37 °C for 30 s. The 2^−ΔΔCt^ method was employed to calculate the relative expression levels of *ZjBURPs* from three biological replicates after normalization to *Zj26S-2* expression (GenBank Accession: NC_029685; forward primer: 5′-TGGCTGAAGAATTGGGCCTT-3′, reverse primer: 5′-AGCCAAAGGAACTGCCCTAC-3′) as the internal reference gene.

## Supplementary Information

**Additional file 1: Fig S1. **The conserved BURP domain and signal peptide of BURP proteins in jujube. The phylogenetic tree of 17 BURP proteins is shown using MEGA-X with the neighbor-joining (NJ) method. The red filled boxed represent the BURP domain, and the green filled boxes represent the signal peptide.

**Additional file 2: Fig S2. **The conserved amino acid residues in BURP domain of jujube sequences. Multialignment of 17 ZjBURP proteins using DNAMAN v. 10.3.3.126. The black-, pink-, and aquamarine-shaded amino acids represented the homology level with 100%, over 75%, and over 50%, respectively

**Additional file 3: Table S1.** Number of cis-elements in putative promoters of ZjBURPs.

**Additional file 4:** **Fig S3. **The original, uncropped gel of RT-PCR analysis of ZjBURPs in three tissues. The red box denoted the region of the original gel. The bands in the F, YF, and L row represented ZjBURPs expression in flower, young fruit, and leaf, respectively. 1-9: ZjBNM1, 2, 3, 4, 5, 6, 7, 8, and 9; 10-14: ZjBURP1, 2, 3, 4, and 5; 15-17: ZjPG1, 2, and 3; 18: Zj26S-2.

**Additional file 5: Table S2.** Primer pairs used in RT-PCR and qRT-PCR analysis.

## Data Availability

The reference jujube genome dataset could be found in the National Center for Biotechnology Information (NCBI) with the accession GCA_001835785.2. All original analysis data have been uploaded as supplementary tables or figures that could be found online. Further detail datasets generated and analyzed could be available from the corresponding author on reasonable request.

## Abbreviations

BURP: BNM2, USP, RD22 and PG1β
FPKM: Fragments per kilobase of transcript per million fragments
qRT-PCR: Quantitative real-time polymerase chain reaction
