# Supplementary material for: Identification and expression analysis of BURP domain-containing genes in jujube and their involvement in low temperature and drought response
Source: BMC Genomics. 2022 Oct 6;23:692. doi: 10.1186/s12864-022-08907-9 (PMC9541082; doi:10.1186/s12864-022-08907-9)
Supplement: Supplementary file 1 — Additional file 1: Fig S1. The conserved BURP domain and signal peptide of BURP proteins in jujube. The phylogenetic tree of 17 BURP proteins is shown using MEGA-X with the neighbor-joining (NJ) method. The red filled boxed represent the BURP domain, and the green filled boxes represent the signal peptide. [file 12864_2022_8907_MOESM1_ESM.pdf]

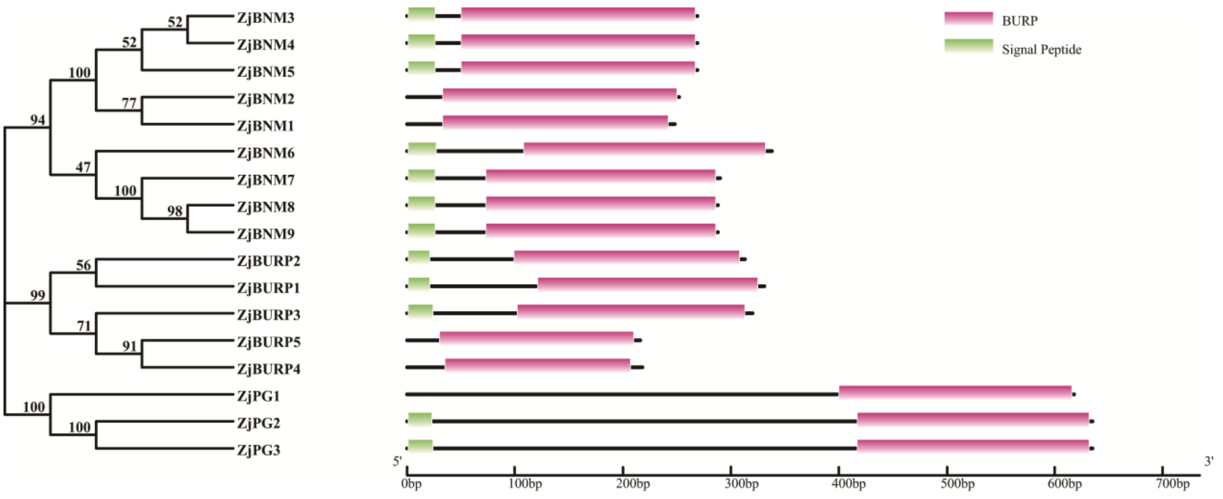

Additional file 1: Fig S1. The conserved BURP domain and signal peptide of BURP proteins in jujube. The phylogenetic tree of 17 BURP proteins is shown using MEGA-X with the neighbor-joining (NJ) method. The red filled boxed represent the BURP domain, and the green filled boxes represent the signal peptide
