# Supplementary material for: Identification and expression analysis of BURP domain-containing genes in jujube and their involvement in low temperature and drought response
Source: BMC Genomics. 2022 Oct 6;23:692. doi: 10.1186/s12864-022-08907-9 (PMC9541082; doi:10.1186/s12864-022-08907-9)
Supplement: Supplementary file 2 — Additional file 2: Fig S2. The conserved amino acid residues in BURP domain of jujube sequences. Multialignment of 17 ZjBURP proteins using DNAMAN v. 10.3.3.126. The black-, pink-, and aquamarine-shaded amino acids represented the homology level with 100%, over 75%, and over 50%, respectively [file 12864_2022_8907_MOESM2_ESM.pdf]

```
ZjBNM4      FFTVDDLYVGKAVGIQFYTRDPSSLPPFLSKEVADRIPFSVKELPLILQLFGMAPGSPEAQLAERTLRTCAEEPIKGEQKTCATSIESFI     90
ZjBNM5      FFTVDDLYVGKAVGIQFYTRDPSSLPPFLSKEVADRIPFSVKELPLLLQLFGMAPGSPEAQLAERTLKTCAEEPIKGEQKTCATSIESFI     90
ZjBNM3      FFTVDDLYVGKAVGIQFYTRDPSSLPPFLSKEVADRIPFSVKELPLILQLFGMAPGSPEAQLAERTLRTCSEEPIKGEQKTCATSVESFI     90
ZjBNM2      FFTVDDLYVGKVVGIQFYTRDPSSLPPFLSKEVADRFPFSVKELPLILQLFGMAPGSPEAQLAERTLRFCADEPIKGEQKTCATSIESFI     90
ZjBNM1      FFTVDDLYVGKVVGIQFYTRDPSSLPPFLSKEVANHFLFSIKELLLILQLFGMALGSLEAQLAERTLRFCAEEPTKGEQKTCATSIESFI     90
ZjBNM8      FFTVDQLYVGKTMPISFPNIDFSSLPRFLPEREADIIPFTSSKLPQLLQFFLFPQESIQANQIEMTLKTCELKPTKGETKFCATSFESLR     90
ZjBNM9      FFTVDQLYVGKTMPISFPNIDFSSLPRFLPEREADIIPFTSSKLPQLLQFFLFPQESIQANQIEMTLKTCELKPTKGETKFCATSFESLR     90
ZjBNM7      FFTVDQLYVGKTMPISFPNIDFSSLPRFLQKREADIIPFTSSKLPQLLQFFSFPQESLQASQIETTLKTCELKPIKGETKFCATSFESLL     90
ZjBNM6      FFTTNDLVVGKTMPIYFPRNDLSASPRLLPREEADSIPFSASQIQYLLDYFSFSKGSPQAKAIEYTLRQCELEPIKGETRFCATSLESML     90
ZjBURP2     FFLQNDLHSGKKMKLHFP..KSTNQAKFLPRKVSESIPFSTTKLPEILNRFGIQPKSVEAETLRQTIEECEQAGIEGEDKYCATSLEAMV     88
ZjBURP1     PVYVGDLHPGSEMNLHFI..KSSNSQTFLPRQVAQSIPFSSNKLPEIYNQFSVVPGSIEAEIIKETIKECENPSTRGEEKYCATSLESMV     88
ZjBURP5     FFFESDLHEGSKMKLSFP..KIKNSAKFLPRQVSESIPFSTTKLPEILAHFSMK......................SKSLQAKAMEDTL     65
ZjBURP4     LFFETYLHEESIPFLNYK..ATGDLTHFSIKPESLEAKAMEDSLKLCEAPAIKC......................EDEYCATSLESLV     65
ZjBURP3     YFLVSDIQQERTVKMLFP..KAMNITKFLPRQVAESIPFSSTKLPEIFAHFAIDPKSLEAKTMEIVLKDCEQPFPEEEERRCVTSFESLV     88
ZjPG2       FFRESELKQGKVMVMPDIR.DRMPKRSFLPRTILSKLPFSTSKISELNEIFDARENSAMQRVITNALTECERSPSRGETKRCVGSLEDMV     89
ZjPG3       FFRESELKQGNVMVMPDIR.DRMPKRSFLPRTILSKLPFSTSKISELNEIFDARENSAMQRVITNALTECERSPSRGETKRCVGSLEDMV     89
ZjPG1       FFRESMLKKGVVMPMPDIR.DRMPKRSFLPRSILSKLPFSTSKISDLKRIFHAGDNSTMEKMMLDALEECERAPSRGETKRCVGSMEDMI     89
Consensus                                                                                 e

ZjBNM4      EFASSMLGGGQYGVDFRAIKTTHLGKPVSVYQNYTFLDIKEVHSP.I..MVACHIMDYPYIVYMCHSQTSR..........VYQIKIVGQ     167
ZjBNM5      EFASSMLGGGQYGVDFRAIKTTHLGKPVSVYQNYTFLDIKEVHSP.I..MVACHIMDYPYIVYMCHSQTSR..........VYQIKIVGQ     167
ZjBNM3      EFASSMLGGGQYGVDFRAIKTTHLGKPVSVYQNYTFLDIKEVHSP.I..MVACHVMDYPYIVYMCHSQTSR..........VYQIKIVGQ     167
ZjBNM2      EFASSMLGGGQYGVDFRAIKTTHLGKPVSVYQNYTFLDIKEVHSP.I..MVACHIMDYPYIVYMCHSQTSR..........VYQIKIVGQ     167
ZjBNM1      EFASSMLGGGQYGVDFRSIKTTHLGKPVSIYQNYTFLDIKEVHSP.I..VVAFHIMDYPYIVYMCHSQTSR..........VYQIKIVGQ     167
ZjBNM8      DFVRDILG...KENHYKVLTSQHIKNP..VAQNYTFLEISQVFSS.SNKIVACHILPYPYAVFYCHSPEAS.........KLFKIRVGGN     165
ZjBNM9      DFVRDVLG...KENHYKVLPTQHIKNP..VAQNYTFLEISQVFSS.SNKIVACHILPYPYAVFYCHSPEAS.........KLFKIRVGGN     165
ZjBNM7      DFVRDVLG...KENHYKVLTTQHIKNP..VAQNYTFLEISQVFSS.SNKIVACHILPYPYAVFYCHSPEAS.........KLFKIRVRGN     165
ZjBNM6      EFARDIFG...FDARFRVLTTTHITNSTILLQNYTILETPKQIFA.P.KMIACHTMPYPYAVFYCHNQESKNNHNQESKNNVFEISLG.S     174
ZjBURP2     EFSVSKLGN.......KNINVLSIESDKGISKQVYSIVDKGVKKI.GDRSVICHKQKYAYAVFYCHEIKAT.........KTYTVSMVGN     161
ZjBURP1     DFSTSKLG........KDALAVSTEVDKETQMQKYTIVPQVKKMV.GDKSVVCHKQNYAYAVFYCHTTEST.........ETFIVPLEGA     160
ZjBURP5     KVCEAPAL.........NGEDKYR....EKIPHVYTIGRGTKMI..GDKSVECHKLNYVYAVFYCHEVQGT.........RAYRVPLKQQ     131
ZjBURP4     DFGVSKLG.........NKIEIFTTGIQEEEPQRYTIGRGTQRI..GDKSVACHKLNYVYAVFYCHDIQDT.........KAYRVPLKMQ     135
ZjBURP3     DFVVSKIG.........NKVDVYVTEVENEEAQEFIIGKKTELKN.GDVSVICHKENFAYAVFYCHKIYGT.........KAYWVPLKKV     159
ZjPG2       DFAVTVLG........RNVVVRTTANVNGSKRKVMIGKVKGVNGGNVTKSVSCHQSLYPYLLYYCHSVPRVR.........VYEADIHDA     162
ZjPG3       DFAVTVLG........RNVVVRTTANVNGSKRNVMIGKVNGVNGGNVTKSVSCHQSLYPYLLYYCHSVPRVR.........VYEADIHDA     162
ZjPG1       DFATSVLG........RNVVVRTTENVNGSKKNILIGNVKGINGGDITQSVSCHQSLFPYLLYYCHSVPKVR.........VYQADLLDP     162
Consensus                                                        h     y    ch

ZjBNM4      EVG......DALNAIAVCHIDTSQWAPDHVSFKLLSVKPGTVSICHFFGPHNPVWVKN....                                219
ZjBNM5      EVG......DALNAIAVCHIDTSQWAPDHVSFKLLGVKPGTVSICHFFGPHNPVWVKT....                                219
ZjBNM3      EVG......DALNAIAVCHIDTSQWAPDHVSFKLLGVKPGTVSICHFFGPHNPVWVKN....                                219
ZjBNM2      EVG......DALNAVAVCHIDTSQWAPDHVSFKLLGVKPGTVSICHFFGPHNPVWVKN....                                219
ZjBNM1      NVG......DALNAFVICHIDTSQWAPDHISFKLLSVKPGTILICHFFGPHNPV........                                215
ZjBNM8      ENG......DKVDGLSVCHMDTSDWISD.DMFTLLGVKRGT.PICHFFSPATLIWVSY....                                215
ZjBNM9      ENG......DKVDGLSVCHMDTSDWISD.DMFILLGVKRGT.PICHFFSPATLIWVSY....                                215
ZjBNM7      ENG......DKVDGLAVCHMDTSDWISD.DMFILLGVKRGN.PICHFFSPATLIWVSYQP..                                217
ZjBNM6      ENG......DRVEAAAVCHMDTSRWDRDHAAFSVLKIEPGTSPVCHFFPADNLVWVPLPAKI                                230
ZjBURP2     DGT.......KAKAVAVCHTDTRTWNHDNFAFQLLKIKPGTVPICHFLNSDTLVWVQTSN..                                214
ZjBURP1     DGT.......KAKAVAVCHKDTSTWNPKHVAFQVLNVKPGTVPVCHYLPEDHIVWVKNQKST                                215
ZjBURP5     LQQPR...EDGDVALAVCHSDTSAWNPKNEAFQQLKVKPGTVPICHFLSSDTLFLVNK....                                186
ZjBURP4     HQQ......EDDVAIAVCHSDTSSWNPKHAAFQQLKVKPGTVPICHFLCSDTLFLVNKLLEL                                191
ZjBURP3     GVDDYGAKISKNFAVAICHTETSSWDPNIVPFQQLKVKPG.TAFCHFIRSDTLLVTVANK..                                218
ZjPG2       MSN.....MKINHGVAICHLDTSSWSPLHGAFVALGSSPGQIEVCHWIFENDMSWTTTD...                                216
ZjPG3       MSN.....MKINHGVAICHLDTSSWSPLHGAFVALGSSPGQIEVCHWIFENDMSWTTTD...                                216
ZjPG1       NSK.....AKINHGVAICHLDTSAWSPTHGSFLALGSGPGRIEVCHWIFENDMTWTIAD...                                216
Consensus                        ch t  w      f  l    g    ch
```

Additional file 2: Fig S2. The conserved amino acid residues in BURP domain of jujube sequences. Multi-alignment of 17 ZjBURP proteins using DNAMAN v. 10.3.3.126. The black-, pink-, and aquamarine-shaded amino acids represented the homology level with 100%, over 75%, and over 50%, respectively
