# Supplementary material for: Identification and expression analysis of BURP domain-containing genes in jujube and their involvement in low temperature and drought response
Source: BMC Genomics. 2022 Oct 6;23:692. doi: 10.1186/s12864-022-08907-9 (PMC9541082; doi:10.1186/s12864-022-08907-9)
Supplement: Supplementary file 4 — Additional file 4: Fig S3. The original, uncropped gel of RT-PCR analysis of ZjBURPs in three tissues. The red box denoted the region of the original gel. The bands in the F, YF, and L row represented ZjBURPs expression in flower, young fruit, and leaf, respectively. 1-9: ZjBNM1, 2, 3, 4, 5, 6, 7, 8, and 9; 10-14: ZjBURP1, 2, 3, 4, and 5; 15-17: ZjPG1, 2, and 3; 18: Zj26S-2. [file 12864_2022_8907_MOESM4_ESM.pdf]

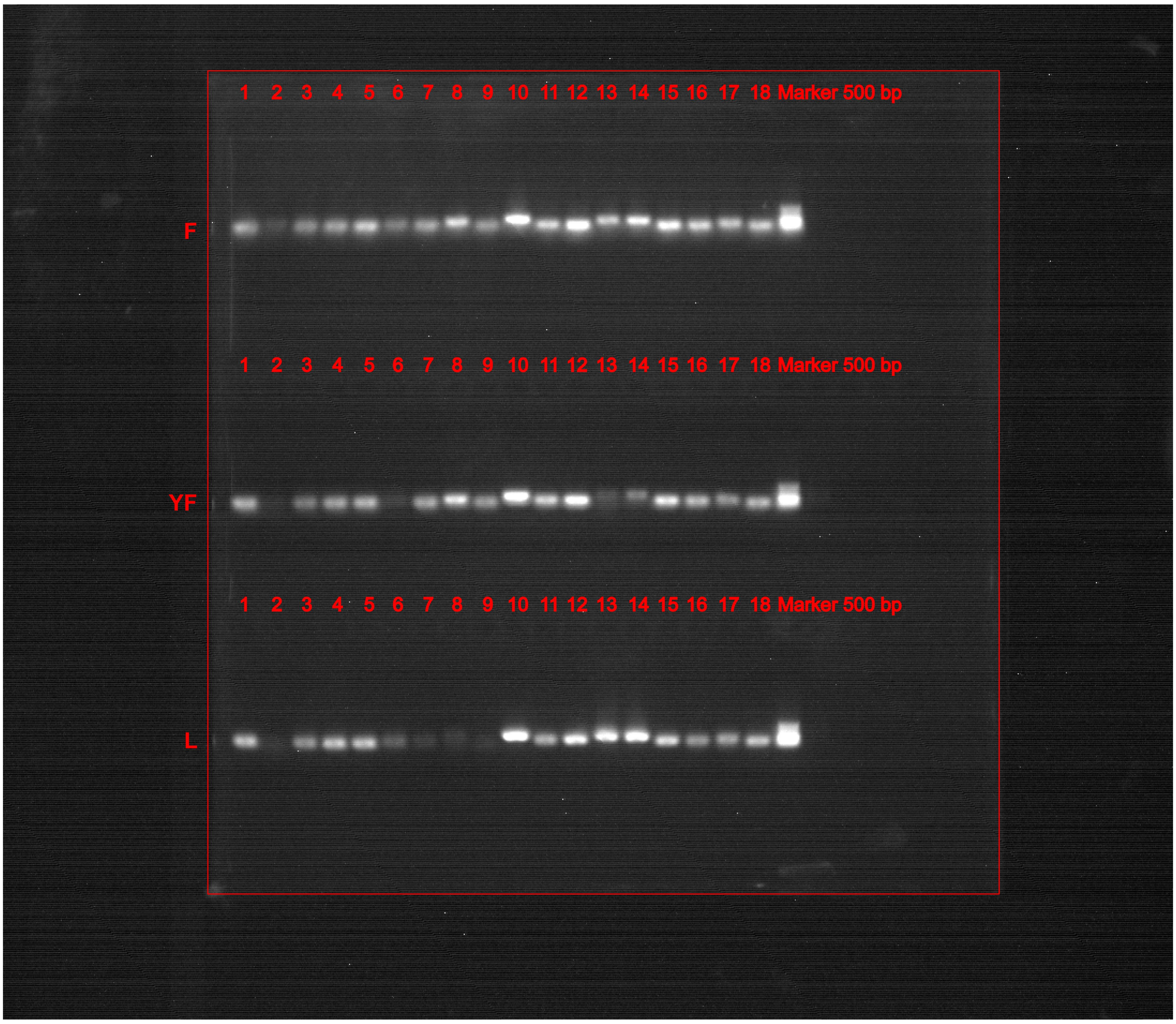

Additional file 4: Fig S3. The original, uncropped gel of RT-PCR analysis of *ZjBURPs* in three tissues. The red box denoted the region of the original gel. The bands in the F, YF, and L row represented *ZjBURPs* expression in flower, young fruit, and leaf, respectively. 1-9: *ZjBNM1*, *2*, *3*, *4*, *5*, *6*, *7*, *8*, and *9*; 10-14: *ZjBURP1*, *2*, *3*, *4*, and *5*; 15-17: *ZjPG1*, *2*, and *3*; 18: *Zj26S-2*.
